# Urbanization, ethnicity and cardiovascular risk in a population in transition in Nakuru, Kenya: a population-based survey

**DOI:** 10.1186/1471-2458-10-569

**Published:** 2010-09-22

**Authors:** Wanjiku Mathenge, Allen Foster, Hannah Kuper

**Affiliations:** 1 London School of Hygiene & Tropical Medicine, Keppel Street, London WC1E 7HT, UK

## Abstract

**Background:**

Cardiovascular disease (CVD) is the leading cause of death among older people in Africa. This study aimed to investigate the relationship of urbanization and ethnicity with CVD risk markers in Kenya.

**Methods:**

A cross-sectional population-based survey was carried out in Nakuru Kenya in 2007-2008. 100 clusters of 50 people aged ≥50 years were selected by probability proportionate to size sampling. Households within clusters were selected through compact segment sampling. Participants were interviewed by nurses to collect socio-demographic and lifestyle information. Nurses measured blood pressure, height, weight and waist and hip circumference. A random finger-prick blood sample was taken to measure glucose and cholesterol levels.

Hypertension was defined as systolic blood pressure (SBP) ≥140 mm Hg, or diastolic blood pressure (DBP) ≥90 mm Hg or current use of antihypertensive medication; Diabetes as reported current medication or diet control for diabetes or random blood glucose level ≥11.1 mmol/L; High cholesterol as random blood cholesterol level ≥5.2 mmol/L; and Obesity as Body Mass Index (BMI)≥30 kg/m^2^.

**Results:**

5010 eligible subjects were selected, of whom 4396 (88%) were examined. There was a high prevalence of hypertension (50.1%, 47.5-52.6%), obesity (13.0%, 11.7-14.5%), diabetes (6.6%, 5.6-7.7%) and high cholesterol (21.1%, 18.6-23.9). Hypertension, diabetes and obesity were more common in urban compared to rural groups and the elevated prevalence generally persisted after adjustment for socio-demographic, lifestyle, obesity and cardiovascular risk markers. There was also a higher prevalence of hypertension, obesity, diabetes and high cholesterol among Kikuyus compared to Kalenjins, even after multivariate adjustment. CVD risk markers were clustered both across the district and within individuals. Few people received treatment for hypertension (15%), while the majority of cases with diabetes received treatment (68%).

**Conclusions:**

CVD risk markers are common in Kenya, particularly in urban areas. Exploring differences in CVD risk markers between ethnic groups may help to elucidate the epidemiology of these conditions.

## Background

Infectious diseases are still the principal cause of death in Africa [1]. However, among older people coronary heart disease (CHD) and stroke are emerging as the leading cause, responsible for more than a quarter of deaths in people 60 years and over in Africa [2,3]. This represents a dramatic shift as CHD was virtually unknown in Africa until recently [4,5]. Stroke is particularly common in Africa in comparison to CHD [6], and stroke mortality rates and prevalence of disabling stroke in most African countries are comparable to levels seen in high-income countries [7-9]. African countries are therefore experiencing a shift in the epidemiological transition [10], while retaining a high burden of infectious diseases.

The rise in cardiovascular disease (CVD) is linked to the increase in hypertension, diabetes, obesity, and high cholesterol observed in Africa in recent years. Obesity [11-14] and hypertension [11,15-17] are now common throughout Africa, particularly in urban areas [11,12,14,16,17]. The number of people with diabetes in Sub-Saharan Africa is expected to more than double between 2000 and 2030 [18], and diabetes is particularly common in urban areas [19]. Urbanisation is therefore a key feature in the rise of CVD. Currently 40% of Africans live in urban areas [20], and it is estimated that by 2030 half of Africans will live in urban areas. The Kenyan Luo Migration Study elegantly demonstrated the impact of rural-urban migration on CVD risk in Africa [21]. Rural migrants to Nairobi experienced a rise in systolic and diastolic blood pressure after only one month of migration. In contrast, the effect of rural-urban migration on blood pressure was not observed in a recent study in Tanzania, even though physical activity levels fell and weight increased after migration [22]. The discrepancy between the two studies may be because the rural-urban contrast in sodium intake was smaller in the Tanzanian compared to the Kenyan study (personal communication). The impact of rural-urban migration on health may therefore vary depending on setting.

People of African origin may be particularly vulnerable to hypertension. The prevalence of hypertension is high among people of African origin compared to Whites independent of BMI [23-25], and there is a younger age at onset in Africa [15] and among people of African descent [26]. Although the same risk markers are largely responsible for myocardial infarction (MI) across the globe [27], hypertension was associated with a higher MI risk in the Black African group than in the overall INTERHEART group [28]. There may also be greater difficulty in achieving control [26], and more aggressive presentation [26,29] and progress [28] of hypertension among people of African origin. This vulnerability may be due to lifestyle factors, but may also be influenced by ethnicity which varies widely within Africa and is linked to substantial heterogeneity in body composition [14], which may exert important metabolic effects [30].

The aim of this study was to investigate the relationship of urbanization and ethnicity with the prevalence of obesity, hypertension, diabetes and high cholesterol in a study of elderly people in Nakuru district, Kenya.

## Methods

### Settings and population

Nakuru district has a population of 1.2 million, one third of which is urban. Nakuru is broadly representative of Kenya in terms of ethnic diversity and economic activities. The two dominant ethnic groups are Kikuyus and Kalenjins. The Kikuyu are related to other Bantu-speaking peoples of East Africa while Kalenjins are of Nilotic origin.

During Jan 2007- Dec 2008, a sample of 100 clusters of 50 people aged ≥50 years were selected across Nakuru district through probability proportionate to size sampling, using the electoral role as the sampling frame. Clusters were classed as "rural" or "urban" using the classification of the district statistical office. Households were selected within clusters through modified compact segment sampling [31]. The village leaders produced a sketch map of the polling area. The polling area was divided into segments each including approximately 50 people aged ≥ 50 years. One segment was chosen at random by drawing lots and all households in the segment were included in the sample sequentially, until 50 people aged ≥50 years were identified. If the segment did not include 50 people aged ≥50 years then another segment was chosen at random and sampling continued.

The enumeration team visited households, assisted by a village guide, and invited all eligible participants aged ≥50 years to the examination clinic which would be held at a convenient place in the cluster over the subsequent two days. Eligible participants were defined as those aged ≥50 years resident in the cluster (i.e. living there at least 6 months per year) who had slept in the house either the night before or were planning on sleeping in the house that night. If an eligible person was absent then the survey team revisited the household at least two times.

### Examination clinic

#### Interviews

Participants were interviewed by trained nurses. Information was collected on demographic data, education and assets (building materials of the house - type of walls, roof, floor and toilet; ownership of household assets - radio, TV, fridge, phone, cupboard, sofa set, sewing machine, table, bicycle and vehicle; animal ownership - cows, sheep/goats). People were asked whether their mother tongue was "Kikuyu", "Kalenjin" or other. For simplicity, people will be classified as "Kikuyu" and "Kalenjin" in the text. Information was also collected on health behaviour (smoking, alcohol use) and health status (diagnosis of diabetes or hypertension, family history and their treatment).

#### Physical examination

A nurse recorded the blood pressure of participants three times on the right arm of the participant, at least five minutes apart after an initial period of five minutes of rest using the Omron digital automatic monitor (model HEM907). A medium cuff size was used (to fit arms 22 to 32 cm). The average of the last two readings was used as the measures of systolic and diastolic blood pressure (to nearest 1 mm Hg). A random finger-prick blood sample was taken to measure glucose (Accutrend GC system) and cholesterol levels (Accutrend GC system). The technical data from the company asserts that precision is <3% for glucose and <5% for cholesterol [32]. Weight was measured to the nearest kg using standard scales to the nearest 0.1 kg (Seca 761 scales) after the participant had removed all heavy clothing and shoes. Height was measured to the nearest cm while the participant stood without shoes using a standardized stadiometer (Leicester Height Measure). For weight and height the average of two readings was recorded. Waist and hip circumferences were measured with a tape. The waist circumference was measured at umbilicus level in mid-expiration to the nearest 0.1 cm. The hip circumference was measured at the point of largest gluteal circumference to the nearest 0.1 cm.

### Training

The examination team received three weeks of training. Inter observer variation (IOV) was assessed during the training weeks at the Nakuru Provincial General Hospital. IOV on anthropometric variables were done by repeat measuring of 50 subjects by the two nurses. The level of agreement required was to the nearest 1 cm for circumferences and height, and to the nearest 0.5 kg for weight. The staff were retrained or replaced if IOV scores indicated poor comparability (kappa < 0.5).

### Statistical analysis

Statistical analyses were undertaken using the SAS statistical package (version 9.2). The four CVD risk markers considered were hypertension, diabetes, high cholesterol and obesity. Hypertension was defined as systolic blood pressure (SBP) was ≥140 mm Hg, or diastolic blood pressure (DBP) ≥90 mm Hg or current use of antihypertensive medication [33]. Diabetes was defined as reported current medication (tablets or insulin) or diet control for diabetes or random blood glucose level ≥11.1 mmol/L [34]. People were categorized as having high cholesterol if their random blood cholesterol level was ≥5.2 mmol/L [35]. Obesity was defined as Body Mass Index (BMI)≥30 kg/m^2 ^[36]. Prevalence estimates for the four CVD risk markers were calculated taking account of the design effect (DEFF) in estimating the confidence intervals. The DEFF was not taken into account for other analyses. A relative index of socio-economic status (SES) was calculated based on building materials of the house, ownership of ten household assets and education status using principal components analysis [37]. The derived index was divided into quartiles from poorest to least poor.

We assessed the association between rural-urban status and, in turn, hypertension, diabetes, high cholesterol and obesity through logistic regression models. The models were adjusted in turn for, a) for age (50-59, 60-69, 70-79, ≥80) and sex, b) age, sex and socio-demographic factors (SES score in quartiles), c) age, sex, BMI (<20, 20-25, >25-30, ≥30), waist hip ratio (WHR - in quartiles), smoking (current, former, never) and alcohol (current, former, never), d) age, sex, diabetes and high cholesterol (as appropriate) and e) fully adjusted model. These models were repeated assessing the association between the four CVD markers and Kikuyu or Kalenjin ethnicity, adjusting for urban status in models b and e. We included an interaction factor in the logistic regression models for ethnicity to assess whether there was an interaction between ethnicity and urban status in the relationship with CVD risk markers. We assessed the proportion of people receiving medical treatment among people who were defined as "hypertensive", and attempted to identify predictors of treatment status through logistic regression models. This was repeated for people with diabetes.

We assessed whether the CVD risk markers were clustered geographically by calculating the DEFF for each of the variables. We assessed whether there was clustering of the CVD risk markers within individuals. To do this, we derived expected frequencies of co-occurrence of risk markers (hypertension, diabetes, high cholesterol and obesity) from none through to four risk markers by combining probabilities, assuming a binomial distribution and independence between them [38]. We estimated observed to expected ratios for all participants and then separately for urban and rural groups and for Kikuyu and Kalenjin groups. We considered that there was clustering if the observed:expected ratios were high for no risk markers, low for one risk marker and high for three or more risk markers. We calculated chi-square statistics with 3 degrees of freedom to test the significance of the overall distribution of expected and observed counts within each group.

### Ethical approval

Ethical approval for this work was granted by the London School of Hygiene & Tropical Medicine and The Kenya Medical Research Institute Ethical Committee and Nakuru District Health Management Team. Informed consent was obtained from the subjects. All people with other treatable conditions were referred for appropriate treatment.

## Results

We examined 4,396 (88%) of the 5,010 people invited. Among those examined 1,437 (33%) lived in urban and 2,959 (67%) in rural areas (Table 1). Urban dwellers were younger, and had higher education levels and asset scores than rural dwellers. They were also more likely to be smokers and obese, than rural participants. Kikuyus made up 63% of the sample and Kalenjins 23% while the remaining 15% consisted of other language speakers. Kikuyus were more likely than Kalenjins to live in urban areas or to be female, and they had higher levels of education and higher SES scores. Kikuyus were less likely to be current smokers or consumers of alcohol. Kikuyus were significantly shorter yet heavier than Kalenjins among both men (166 cm vs 168 cm, 64 kg vs 60 kg) and women (156 cm vs 158 cm, 62 kg vs 58 kg) and consequently had higher BMIs.

**Table 1:** Demographic characteristics and health behavior comparing urban and rural participants, and Kikuyus and Kalenjins

	Urban (n = 1437)	Rural (n = 2959)	Age and sex adjusted OR (95% CI)	Kikuyu (n = 2760)	Kalenjin (n = 1015)	Age and sex adjusted OR (95% CI)
Age						
50-59	57%	38%	Baseline	40%	40%	Baseline
60-69	26%	31%	0.6 (0.5-0.7)	32%	28%	1.2 (1.0-1.4)
70-79	11%	20%	0.4 (0.3-0.5)	17%	20%	0.9 (0.7-1.1)
≥80	6%	11%	0.4 (0.3-0.5)	10%	11%	0.9 (0.7-1.2)
Sex						
Men	49%	47%	Baseline	44%	53%	Baseline
Women	51%	53%	0.9 (0.8-1.0)	56%	47%	1.4 (1.2-1.6)
Language						
Kikuyu	65%	62%	Baseline			
Kalenjin	6%	31%	0.2 (0.1-0.2)			
Other	29%	7%	3.3 (2.7-4.0)			
Urban				931 (34%)	84 (8%)	Baseline
Rural				1812 (66%)	924 (92%)	5.7 (4.5-7.2)
Education						
Any	79%	61%	1.4 (1.3-1.6)	71%	51%	1.9 (1.7-2.1)
None	21%	39%	Baseline	29%	49%	Baseline
SES score						
1 (poorest)	8%	33%	Baseline	20%	44%	Baseline
2	14%	31%	1.6 (1.2-2.0)	26%	27%	2.4 (2.0-2.9)
3	24%	26%	3.1 (2.5-3.9)	27%	20%	3.4 (2.8-4.2)
4 (richest)	54%	11%	16.7 (13.3-21.1)	26%	9%	7.8 (6.0-10.2)
Smoking						
Never	70%	70%	Baseline	69%	73%	Baseline
Current	7%	8%	1.4 (1.1-1.8)	8%	6%	0.5 (0.3-0.6)
Former	23%	22%	1.1 (0.9-1.3)	24%	20%	2.0 (1.6-2.5)
Alcohol						
Never	41%	38%	Baseline	45%	23%	Baseline
Former	41%	46%	0.9 (0.7-1.0)	44%	49%	0.4 (0.3-0.5)
Current	18%	17%	0.9 (0.7-1.1)	11%	28%	0.2 (0.1-0.2)
BMI cat						
Underweight	8%	17%	0.7 (0.5-0.9)	12%	21%	0.7 (0.6-0.9)
Normal	41%	54%	Baseline	48%	56%	Baseline
Overweight	30%	19%	2.1 (1.7-2.4)	25%	15%	1.9 (1.5-2.3)
Obese	20%	10%	2.8 (2.3-3.5)	14%	8%	2.0 (1.5-2.6)

**Figure 1:**
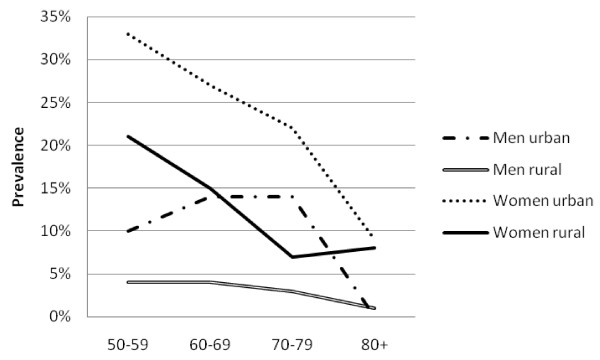
**Age trends in obesity by urban status**.

**Figure 2:**
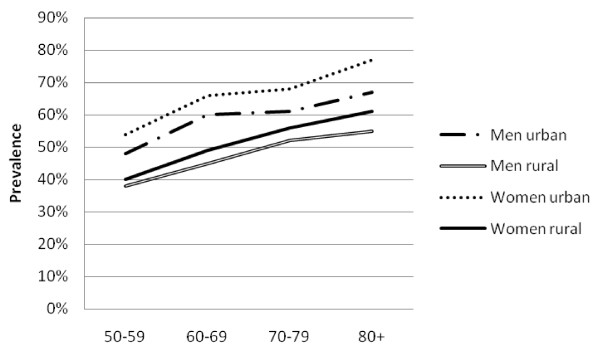
**Age trends in hypertension by urban status**.

**Figure 3:**
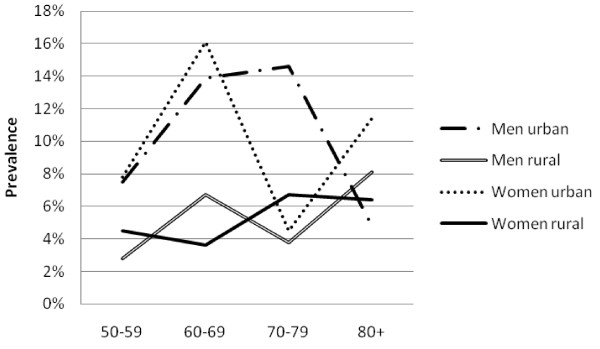
**Age trends in diabetes by urban status**.

**Figure 4:**
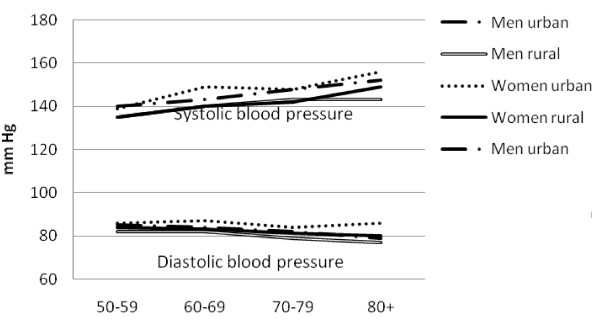
**Age trends in systolic and diastolic blood pressure by urban status**.

**Figure 5:**
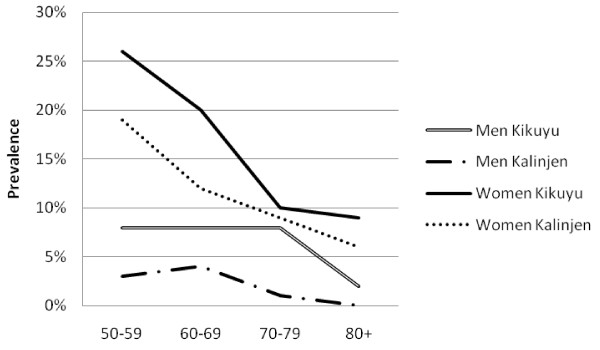
**Age trends in obesity by ethnicity**.

**Figure 6:**
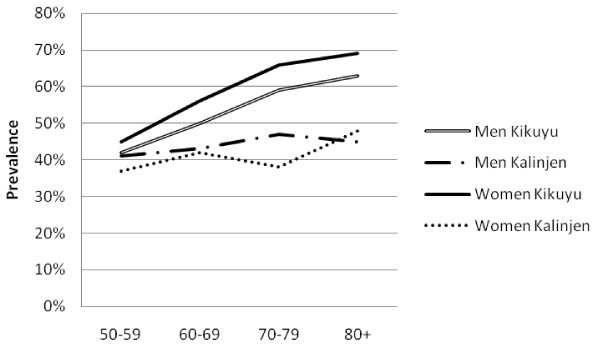
**Age trends in hypertension by ethnicity**.

**Figure 7:**
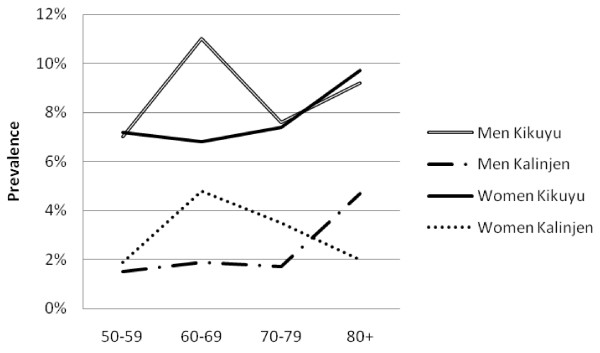
**Age trends in diabetes by ethnicity**.

**Figure 8:**
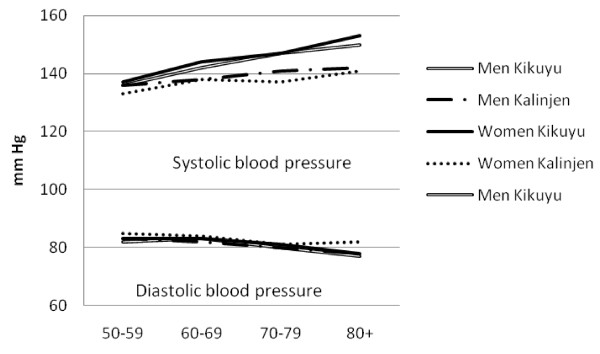
**Age trends in systolic and diastolic blood pressure by ethnicity**.

There was a high prevalence of hypertension (50.1%, 47.5-52.6%), obesity (13.0%, 11.7-14.5%), diabetes (6.6%, 5.6-7.7%) and high cholesterol (21.1%, 18.6-23.9%). Mean SBP and DBP were higher in urban than rural areas among both men and women (Table 2). Similarly, mean glucose and cholesterol levels and markers of obesity were higher in urban than rural men and this pattern was generally repeated among women. The prevalence of obesity generally fell sharply with age (Figure 1). The prevalence of hypertension increased steadily with age, and was consistently higher in urban than rural areas (Figure 2). The association between prevalence and age were less clear for the diabetes (Figure 3) and high cholesterol (data not shown).  Meanwhile, SBP increased with age while DBP decreased, and both remained higher for people from urban than rural areas across the age groups (Figure 4). Kikuyus had higher SBP than Kalenjins, but the differences were less clear for DBP (Table 3). Kikuyus also had higher levels of glucose, cholesterol, BMI and waist circumference but not of WHR. Kikuyus were more likely to be hypertensive, obese or diabetic in all age groups (Figure 5, 6, 7, 8).

**Table 2:** Means (and standard error) of cardiovascular risk markers, by gender and urban-rural status

	Urban-rural comparison							
	Men				Women			
	No. Urban/Rural	Urban	Rural	Age adjusted p-value	No. Urban/Rural	Urban	Rural	Age adjusted p-value
Mean SBP	705/1395	143 (24)	140 (24)	<0.0001	726/1550	143 (26)	140 (25)	<0.0001
Mean DBP	705/1395	84 (14)	81 (13)	<0.0001	726/1550	86 (14)	83 (13)	<0.0001
Glucose	692/1373	5.8 (2.9)	4.8 (2.0)	<0.0001	704/1527	5.7 (2.8)	5.1 (2.3)	<0.0001
Cholesterol	698/1342	4.5 (0.9)	4.3 (0.9)	0.0004	718/1514	4.8 (0.9)	4.7 (1.0)	0.08
BMI	700/1385	24 (6)	22 (4)	<0.0001	718/1543	27 (6)	24 (6)	<0.0001
Waist	703/1390	92 (13)	86 (11)	<0.0001	719/1546	96 (13)	89 (13)	<0.0001
WHR	703/1390	0.92 (0.07)	0.92 (0.06)	0.0009	718/1546	0.89 (0.06)	0.89 (0.08)	0.34

**Table 3:** Means (and standard error) of cardiovascular risk markers, by gender and ethnicity

	Kikuyu-Kalenjin comparison							
	Men				Women			
	No. Kikuyu/Kalenjin	Kikuyu	Kalenjin	Age adjusted p-value	No. Kikuyu/Kalenjin	Kikuyu	Kalenjin	Age adjusted p-value
Mean SBP	1218/535	142 (24)	139 (23)	0.007	1531/473	142 (26)	136 (24)	<0.0001
Mean DBP	1218/535	81 (13)	81 (13)	0.85	1531/473	84 (14)	82 (13)	0.02
Glucose	1209/515	5.5 (2.6)	4.4 (1.7)	<0.0001	1516/449	5.5 (2.6)	4.9 (2.0)	<0.0001
Cholesterol	1177/524	4.4 (1.0)	4.2 (0.7)	<0.0001	1496/466	4.8 (1.0)	4.5 (1.0)	<0.0001
BMI	1207/534	23 (5)	21 (4)	<0.0001	1519/469	26 (6)	23 (5)	<0.0001
Waist	1213/535	89 (12)	86 (11)	0.002	1522/470	91 (14)	88 (13)	<0.0001
WHR	1213/535	0.92 (0.07)	0.92 (0.06)	0.24	1521/470	0.88(0.08)	0.90 (0.06)	<0.0001

The odds of hypertension was higher in urban than rural groups after adjustment for age and sex (Odds ratio - OR = 1.7, 95% CI = 1.5-1.9) (Table 4). The association was weakened after adjustment for SES markers and for obesity, smoking and alcohol, but not after adjustment for diabetes and cholesterol. In the fully adjusted model the odds of hypertension remained higher among urban compared to rural dwellers (1.3, 1.1-1.5). People in urban areas were also more likely to have diabetes (2.3, 1.8-2.9). The increased odds was reduced after adjustment for socio-demographic variables, though adjustment for other CVD risk markers had less effect. In the fully adjusted model the odds of diabetes was no longer elevated in urban compared to rural participants (1.3, 0.9-1.7). No clear association was apparent between the odds of high cholesterol and urban status. The pattern for the association between obesity and urban residence was similar to that for diabetes.

**Table 4:** Rural-urban differences in the prevalence of CVD risk markers

	Urban (n = 1431)	Rural (n = 2945)	Age and sex adjusted OR (95% CI)	Age, sex and socio-demographic adjusted OR (SES score)	Age, sex, BMI, WHR, smoking, alcohol	Age, sex, diabetes, cholesterol, hypertension	Fully adjusted model
Hypertension	812 (57%)	1379 (47%)	1.7 (1.5-1.9)	1.4 (1.2-1.6)	1.4 (1.2-1.6)	1.6 (1.4-1.8)	1.3 (1.1-1.5)
Normotensive	619 (43%)	1566 (53%)	Baseline	Baseline	Baseline	Baseline	Baseline
Diabetic	139 (10.0%)	144 (5.0%)	2.3 (1.8-2.9)	1.3 (1.0-1.7)	2.0 (1.5-2.5)	2.2 (1.7-2.8)	1.3 (0.9-1.7)
Normal	1256 (90.0%)	2756 (95.0%)	Baseline	Baseline	Baseline	Baseline	Baseline
High cholesterol	316 (22%)	588 (21%)	1.2 (1.0-1.4)	0.9 (0.8-1.1)	1.0 (0.9-1.2)	1.1 (0.9-1.3)	0.9 (0.7-1.1)
Normal cholesterol	1100 (78%)	2268 (79%)	Baseline	Baseline	Baseline	Baseline	Baseline
Obese	287 (20%)	280 (10%)	2.3 (1.9-2.8)	1.3 (1.0-1.6)	2.9 (2.3-3.5)	2.2 (1.8-2.7)	1.5 (1.2-1.9)
Not obese	1131 (80%)	2648 (90%)	Baseline	Baseline	Baseline	Baseline	Baseline

The odds of hypertension was higher among Kikuyus compared to Kalenjins (1.6, 1.4-1.8) (Table 5). The association persisted after adjustment for socio-demographic variables and other CVD risk markers (1.4, 1.2-1.7). Similarly, Kikuyus were more likely to have diabetes and high cholesterol, and these associations were not fully explained by adjustment for potential confounders. Kikuyus were more likely to be obese compared to Kalenjin, but not in models adjustment for SES and urban status. For all four risk markers, the biggest change in the association occurred after adjustment for SES and urban status.

**Table 5:** Kikuyu-Kalenjin differences in the prevalence of CVD risk markers

	Kikuyus	Kalenjins	Age and sex adjusted OR (95% CI)	Age, sex and socio-demographic adjusted OR (SES score and urban)	Age, sex, BMI, WHR, smoking, alcohol	Age, sex, diabetes, cholesterol, hypertension	Fully adjusted model
Hypertension	1445 (53%)	420 (42%)	1.6 (1.4-1.8)	1.3 (1.1-1.5)	1.5 (1.3-1.8)	1.5 (1.3-1.7)	1.4 (1.2-1.7)
Normotensive	1304 (47%)	588 (58%)	Baseline	Baseline	Baseline	Baseline	Baseline
Diabetic	219 (8%)	24 (2%)	3.4 (2.2-5.3)	2.2 (1.4-3.4)	3.4 (2.2-5.4)	3.3 (2.2-5.2)	2.3 (1.5-3.8)
Normal	2506 (92%)	940 (98%)	Baseline	Baseline	Baseline	Baseline	Baseline
High cholesterol	648 (24%)	142 (14%)	1.8 (1.5-2.2)	1.6 (1.3-2.0)	1.6 (1.3-2.0)	1.7 (1.4-2.1)	1.5 (1.2-1.9)
Normal cholesterol	2025 (76%)	848 (86%)	Baseline	Baseline	Baseline	Baseline	Baseline
Obese	392 (14%)	80 (8%)	1.8 (1.4-2.3)	1.0 (0.8-1.3)	2.0 (1.5-2.7)	1.7 (1.3-2.2)	1.0 (0.7-1.4)
Not obese	2334 (86%)	923 (92%)	Baseline	Baseline	Baseline	Baseline	Baseline

There was no interaction between urban status and ethnicity for these conditions after adjustment for age and sex (data not shown).

There was substantial variation in the prevalence of hypertension (range 17-77% (DEFF = 2.9) diabetes (range = 0-26%; DEFF = 1.8), high cholesterol (range 0-51.2%, DEFF = 4.3) and obesity (range 0-40%, DEFF = 1.8) between clusters. The variation was similar in rural and urban areas and among Kikuyus and Kalenjins.

Few people had 3-4 (4.5%) or 2 (18.5%) risk markers and the vast majority of the population had no (37.0%) or one (40.1%) risk marker (Table 6). Generally rural dwellers and Kalenjins had fewer risk factors than urban dwellers and Kikuyus. There was a greater than expected frequency of people with 3-4 risk markers than would be expected by chance, and to a lesser extent a higher frequency of people with no risk markers, except among Kalenijn. Together, this provides evidence for clustering of risk markers within individuals, which was more apparent among rural versus urban dwellers, and among Kalenjins compared to Kikuyus.

**Table 6:** Clustering of risk markers by rural-urban status and ethnicity

No. of risk markers*	Total group			Urban			Rural			Kikuyu			Kalenjin		
	Exp**	**Obs**	**O:E**	Exp**	**Obs**	**O:E**	Exp**	**Obs**	**O:E**	Exp**	**Obs**	**O:E**	Exp**	**Obs**	**O:E**

0	32.0	37.0	116	24.1	30.0	124	36.3	40.4	111	28.3	33.1	117	44.8	47.0	105
1	47.7	40.1	84	47.4	38.6	81	47.1	40.9	87	47.6	40.3	85	44.6	40.8	92
2	17.9	18.5	103	23.8	24.9	104	15.0	15.3	102	20.8	21.3	102	9.9	10.7	108
3-4	2.3	4.5	193	4.4	6.5	148	1.5	3.5	228	3.2	5.2	165	0.7	1.6	233
															
Χ^2 ^(3 df)	71.6 P < 0.0001			27.0 p < 0.0001			39.3 p < 0.0001			40.3 p < 0.0001			6.2 p = 0.10		

*Hypertension, obesity, diabetes and high cholesterol

**Based on random assortment of four risk marker

Among people with hypertension, only 323 (15%) received drug treatment, 38 (2%) received diet treatment and 7 traditional medicine treatment (0.3%) (Table 7). Among those on drug treatment, only 98 (29%) had controlled hypertension. A far higher proportion of people with diabetes were receiving treatment (68%), which included insulin (n = 32), tablets (n = 143) and/or diet control (n = 43). A further 10 received traditional treatment. For both hypertension and diabetes, treatment was more among people living in urban areas, women, older people and those with a higher SES score. Kalenjins were less likely to receive treatment for hypertension than Kikuyus (0.5, 0.3-0.8), but there was no difference for diabetes.

**Table 7:** Treatment for hypertension and diabetes

	Hypertension					Diabetes		
	Drug treatment *	No treatment	% untreated	Multivariate adjusted OR (95% CI)***	Treatment**	No treatment	% untreated	Multivariate adjusted OR (95% CI) ***
Number	323	1868			196	91		
Rural	191	621	76%	Baseline	86	59	41%	Baseline
Urban	132	1247	90%	1.6 (1.2-2.1)	110	32	23%	2.0 (1.1-3.8)
Language								
Kikuyu	232	1213	84%	Baseline	152	69	31%	Baseline
Kalenjin	20	400	95%	0.5 (0.3-0.8)	15	10	40%	0.9 (0.3-2.1)
Other	72	255	78%	1.3 (0.9-1.8)	29	12	29%	0.9 (0.4-2.0)
Age								
50-59	128	726	85%	Baseline	67	40	37%	Baseline
60-69	107	561	84%	1.7 (1.2-2.3)	74	29	28%	1.9 (1.0-3.5)
70-79	57	357	86%	2.0 (1.4-3.0)	34	12	26%	3.3 (1.4-7.9)
≥80	31	224	88%	2.0 (1.2-3.3)	21	10	32%	2.6 (0.9-7.3)
Male	93	923	91%	Baseline	92	47	34%	Baseline
Female	230	945	80%	3.4 (2.5-4.5)	104	44	30%	1.8 (1.0-3.1)
SES score								
1 (poorest)	23	456	95%	Baseline	14	11	44%	Baseline
2	39	456	92%	1.4 (0.8-2.3)	19	22	54%	0.7 (0.3-2.1)
3	87	493	85%	2.7 (1.7-4.4)	60	24	29%	2.3 (0.9-5.9)
4 (richest)	169	456	73%	4.7 (2.9-7.7)	102	33	24%	2.2 (0.8-5.8)
Schooling								
Any	249	1217	83%	1.3 (1.1-1.5)	41	24	37%	1.2 (0.8-1.8)
None	74	651	90%	Baseline	155	67	30%	Baseline

*Treatment for hypertension included drug treatment only

**Treatment for diabetes included insulin, tablets or diet control

*** Adjusted for age, sex, urban/rural, ethnic group, SES score, schooling.

### Sensitivity analysis

We assessed the impact of lowering the threshold random blood glucose level for the classification of "diabetes". Lowering the threshold to ≥10 mmol/L added an additional 13 cases to the original 283 cases (revised prevalence 6.9%), for ≥9 mmol/L this was 41 cases (7.5%), for ≥8 mmol/L this was 84 cases (8.5%) and for ≥7 mmol/L this was 180 cases (10.8%). At the least conservative threshold for diabetes (≥7 mmol/L) almost half of cases (42%) were receiving treatment.

## Discussion

This large survey in Kenya highlighted the high prevalence of CVD risk markers, particularly in urban areas. SES was a more important mediator of the association between the individual CVD risk markers and urban status than health behavior or other CVD markers. However, the urban-rural differences in hypertension and obesity were not explained fully after adjustment for SES, obesity, smoking, alcohol or other CVD risk markers. The prevalence of CVD risk markers was higher among Kikuyus than among Kalenjins. Again, these associations were not fully explained after adjustment for the possible confounders, including urban status. A high degree of clustering of these risk markers was apparent, both geographically and within individuals. The clustering within individuals was more marked among rural dwellers and Kalenjins, although they had fewer people with risk factors overall, potentially indicating that there were a few early adopters of these multiple risk factors compared to the more well established presence among urban dwellers and Kikuyus. Only 15% of people with hypertensive were receiving treatment, and this was particularly low among poorer people or rural dwellers. In contrast, more than two thirds of people with diabetes were receiving treatment, although this proportion fell if a lower blood glucose threshold was used for diagnosis of diabetes.

The urban-rural differences in CVD risk markers are likely to be explained by differences in health behavior, including diet and physical activity. These urban-rural differences in lifestyle may in turn explain some of the differences in CVD risk markers between Kikuyus and Kalenjins. However, Kikuyu and Kalenjin participants clearly differed in physical characteristics, such as height, weight and waist and hip circumferences, and these may exert metabolic consequences [30], and explain some of the differences in CVD risk markers.

The prevalence of hypertension in men in our survey was generally high compared to other world regions, and exceeded the prevalence in the Established Market Economies and Latin American Countries for the oldest age group [15]. For women in our survey the pattern was more typical to that seen in the sub-Saharan Africa region. Other studies from Africa confirm the higher prevalence of hypertension, diabetes and obesity in urban compared to rural populations [11,12,14,16,17,19,39]. Previous surveys confirm our finding of a higher prevalence of obesity among women compared to men in Africa [12-14], and suggest that BMI differences between rural and urban areas drive differences in diabetes prevalence [39]. Few people with hypertension were currently treated in the Nakuru survey, which is consistent with the findings from other African settings [7,17,40], while the proportion of people with diabetics receiving treatment was higher than in other surveys [39].

The high prevalence of CVD markers in Nakuru, particularly hypertension, is likely to be of substantial public health importance, as untreated hypertension is an important modifiable risk factor for stroke in Africa [7,41]. This is compounded by the low proportion of hypertensives receiving treatment in this population, although the situation was better for diabetes. The rise of non communicable diseases are likely to put further pressure on an already overstretch primary health care system [9], and so prevention is important potentially through reducing obesity and salt intake [23,42,43]. Further studies investigating means of reducing hypertension in Africa are needed urgently [44].

There were a number of limitations to this study. The study design was a cross-sectional survey, and so we could not take account of the temporal relationship between potential risk markers and outcomes. We measured blood pressure on only one day and so regression to the mean was possible, and one cuff size was used for all participants. The blood glucose measures were obtained from non-fasting blood samples, rather than through use of the oral glucose tolerance test or fasting blood glucose, and this may have underestimated the prevalence of diabetes. Lowering the threshold blood glucose level cut-off for the definition of diabetes increased the prevalence, however, this was not substantial until the level was reduced to >7 mmol/L. We measured smoking and alcohol status, but we did not assess physical activity or diet or other blood markers (e.g. renin), which were potentially important explanatory variables. Although we measured BMI and WHR, it would have been useful to include bioimpedance as a measure of body fat. Classifying people as "Kikuyu" or "Kalenjin" on the basis of their mother tongue may have been over-simplistic. This study also had important strengths. There was a high response rate, and the sample was representative across Nakuru, limiting the impact of selection bias. We included a measure of SES which was previously validated for this area [37]. The measures of blood pressure and anthropometry were assessed by trained medical staff.

## Conclusions

The burden of CVD risk markers is high in Kenya, particularly in urban areas. Exploring differences in CVD risk markers between ethnic groups may help us to elucidate the epidemiology of these conditions in Africa.

## Abbreviations

BMI: Body Mass Index; CHD: Coronary heart disease (CHD); CVD: Cardiovascular disease; DBP: Diastolic blood pressure; DEFF: Design effect; IOV: Inter observer variation; MI: Myocardial infarction; SBP: Systolic blood pressure; SES: Socio-economic status; WHR: Waist hip ratio.

## Competing interests

The authors declare that they have no competing interests.

## Authors' contributions

WM was responsible for carrying out the fieldwork and the cleaning and preparation of the database. HK and AF assisted with the design and supervision of the fieldwork. HK was primarily responsible for the data analysis and producing the first draft of the paper. All authors read and approved the final manuscript.

## Pre-publication history

The pre-publication history for this paper can be accessed here:

http://www.biomedcentral.com/1471-2458/10/569/prepub
